# Immunosuppressive Extracellular Vesicles as a Linking Factor in the Development of Tumor and Endometriotic Lesions in the Gynecologic Tract

**DOI:** 10.3390/cells11091483

**Published:** 2022-04-28

**Authors:** Karolina Soroczynska, Lukasz Zareba, Magdalena Dlugolecka, Malgorzata Czystowska-Kuzmicz

**Affiliations:** 1 Chair and Department of Biochemistry, Medical University of Warsaw, Banacha 1 St., 02-097 Warsaw, Poland; karolina.soroczynska@wum.edu.pl (K.S.); lzareba@wum.edu.pl (L.Z.); magdalena.dlugolecka@wum.edu.pl (M.D.); 2 Postgraduate School of Molecular Medicine, Medical University of Warsaw, Zwirki i Wigury 61 St., 02-091 Warsaw, Poland; 3 Chair and Department of Biochemistry, Doctoral School, Medical University of Warsaw, Zwirki i Wigury 61 St., 02-091 Warsaw, Poland

**Keywords:** extracellular vesicles, immunosuppression, chronic inflammatory microenvironment, tumor microenvironment, pro-endometriotic niche, immune dysfunction, endometriosis, ovarian cancer, endometrial cancer

## Abstract

Both gynecological tumors and endometriosis require for their development a favorable environment, termed in the case of tumors a “pre-metastatic niche” and in case of endometriosis a “pro-endometriotic niche”. This is characterized by chronic inflammation and immunosuppression that support the further progression of initial lesions. This microenvironment is established and shaped in the course of a vivid cross-talk between the tumor or endometrial cells with other stromal, endothelial and immune cells. There is emerging evidence that extracellular vesicles (EVs) play a key role in this cellular communication, mediating both in tumors and endometriosis similar immunosuppressive and pro-inflammatory mechanisms. In this review, we discuss the latest findings about EVs as immunosuppressive factors, highlighting the parallels between gynecological tumors and endometriosis. Furthermore, we outline their role as potential diagnostic or prognostic biomarkers as well as their future in therapeutic applications.

## 1. Introduction

Cancer and endometriosis, although at first glance to be quite different diseases, possess strikingly similar features and underlying mechanisms. The “seed-and-soil” hypothesis proposed by Paget in 1889 regarding tumor metastasis [1], and Sampson’s theory on the pathogenesis [2], pre-suppose similar pre-conditions of disease development: innate or acquired survival of tumor or ectopic endometrial cells, respectively, and an active crosstalk between these cells (the ‘seed’) and other tissues—aimed at the establishment of a suitable microenvironment for the implantation of tumor or endometrial cells (the “soil”) [3]. This favorable environment, also known as the pre-metastatic niche—or pre-endometriotic niche in case of endometriosis, is mediated by chronic inflammation and the dysregulation of multiple signaling pathways, affecting cell proliferation and invasion, angiogenesis, stromal cell remodeling and immune clearance and defense. Despite this highly inflammatory environment, studies have also shown an increase of anti-inflammatory cytokines, starting with the TGFβ family, in endometriosis, leading to a co-existence of high concentrations of pro- and anti-inflammatory cytokines.

Higher levels of TGFβ have been noted in peripheral blood, ectopic endometrium and peritoneal fluid of patients with endometriosis. Nearly all cells present in the endometriotic milieu were shown to produce TGFβ-1, such as endometrial epithelial cells, peritoneal mesothelial cells, endometrial mesenchymal stem cells, endometrial stromal cells, platelets, macrophages and Tregs, as reviewed in [4]. In the case of ovarian cancer (OvCa), the tumor cells acquire resistance to the growth inhibitory effects of TGFβ by loss of c-myc repression and switch to a tumor-promoting TGFβ signaling pathway. Hereby, TGFβ changes its role from tumor suppressor in normal cells to tumor promoter in cancer, favoring invasiveness and metastasis [5]. Up-regulated TGFβ activity has been repeatedly observed in OvCa tumors [6]. Although many aspects of endometriosis onset and development still remain unclear, continuous research advances highlight the critical role of inflammation and TGFβ in growth, cell adhesion, endometrial epithelial-mesenchymal transition (EMT) and invasion, angiogenesis, fibrogenesis and immune escape, both in OvCa and endometriosis [7].

In recent years, extracellular vesicles (EVs) released by tumor cells and other cells in the tumor-microenvironment have been shown to be major factors that promote pre-metastatic niche formation and metastasis, tumor immune escape and chemotherapeutic resistance in several cancer types, including OvCa and other gynecological malignancies [8]. However, given the parallels in the pathogenesis of cancer and endometriosis, it is not surprising that a similar role of EVs has been recently discovered for endometriosis. Evidence is accumulating that, analogous to the key role of EVs in pre-metastatic niche formation and metastatic organotropism in cancers [9], EVs in the uterine and peritoneal microenvironment also may support implementation and growth of ectopic endometrial cells outside the uterus. 

**Figure 1:**
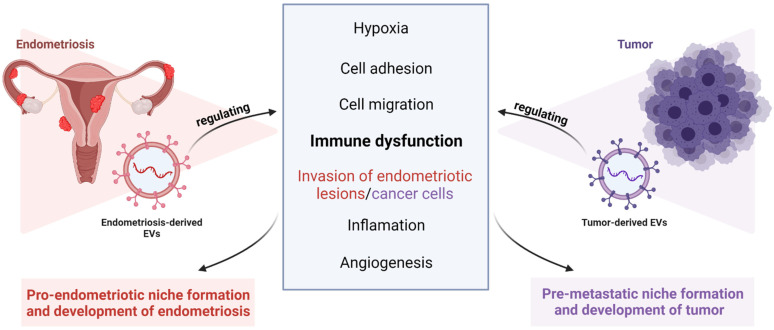
Extracellular vesicles (EVs) derived from diverse microenvironments of endometriosis and gynecologic tract malignancies mediate similar disease progression mechanisms [10]. In this review, we focus exclusively on EV-mediated immune suppression activities. This image was created using BioRender (http://biorender.com/ accessed on 22 April 2022).

Since the role of EVs in angiogenesis and neurogenesis, migration and invasion during niche formation and metastasis and therapy resistance has been extensively discussed elsewhere, mainly in the context of cancer, but also in endometriosis [10], we focus in this review mainly on the role of EVs in immunosuppression and the mediation of an inflammatory microenvironment in both diseases (Figure 1). First, we discuss the well acknowledged role of EVs in tumor-immunosuppression, focusing on OvCa and other gynecological tumours. Then, we summarize the latest literature supporting the idea that endometriosis may be a malignant precursor and that especially clear cell and endometrioid carcinoma derive from ovarian endometriosis. Next, we describe the latest findings showing that EVs mediate similar immunosuppressive mechanisms in endometriosis, as in OvCa, and may be responsible for the observed dysfunction of several types of immune cells in this disease. We also discuss the therapeutic potential of EVs for cancer and endometriosis treatment.

## 2. Biogenesis, Components and Characterization of Extracellular Vesicles

Extracellular vesicles communicate with target cells through many different pathways. These mechanisms include fusion, phagocytosis, ligand-receptor interaction, and proteolytic cleavage [11]. The EVs can be divided into a few different types depending on their size, biogenesis process, or their function [12]. Exosomes are one type of EV broadly examined by researchers all over the world. This type of EV is produced in a multi-step process. Their biogenesis begins with endocytosis and the formation of early endosomes. Then, endosomes migrate deep inside the cell, and inward budding of their membrane occurs. The cell lumen proteins, nucleic acids, and lipids are locked inside small spherical membrane intraluminal vesicles (ILVs) which are the exosome precursors. The endosomes with ILVs are called multivesicular bodies (MVBs). Then, the MVBs migrate to the cell surface. Their membrane connects to the cell membrane and their cargo is released by exocytosis into the extracellular space. Released vesicles become exosomes [13]. Exosomes are spherical-shaped vesicles of 30–150 nm, they have a double bilayer phospholipid membrane and contain the cargo from the cell of origin [14]. Existing separation methods are not able to accurately separate exosomes from other small EVs (sEVs), and “true” exosomes can be defined only by their biogenesis. In light of the isolation procedure, almost all published literature, while frequently referring to exosomes, really refers to sEVs rather than exosomes. Therefore, for simplicity we use the term EVs throughout our review, regardless of the nomenclature used in the cited articles, to make it more consistent and clearer for the reader. The decision about the isolation technique is crucial, depends on the desired balance between the purity and yield of the isolated sEVs and the downstream applications and must be made with caution. The correct usage of EV nomenclature and recommendations regarding EV analysis are described in detail in *The first Minimal Information for Studies of Extracellular Vesicles* (MISEV) guidelines [15,16]. 

Extracellular vesicles are found in almost all body fluids and are secreted by all cell types in both healthy and pathological conditions [17]. They carry a variety biologically active molecules as cargo, including proteins, lipids, DNA, and RNAs, which contribute to cellular communication and participate in a number of physiological and pathological processes [18], including those that occur in the female reproductive tract Indeed, it has been reported that EVs secreted by reproductive cells such as endometrial cells, follicular cells, embryos produced in vitro, and oviductal cells released from different parts of female reproductive tract, play a remarkable role in female gynecologicall diseases, as well as in reproductive physiology, as reviewed in [19]. Alterations in EV content, particularly regarding aberrant microRNA expression, have been found in the majority of female gynecologicaldiseases, including OvCa, endometrial cancer, and endometriosis, which are the main focus of this review, as well as in other diseases such as polycystic ovary syndrome (PCOS), premature ovarian failure (POF), Asherman syndrome, cervical cancer, and preeclampsia, as reviewed in [19]. Moreover, changes in the miRNA levels observed during these pathological conditions have been attributed to multiple signaling pathways related to the regulation of the reproductive system and pregnancy outcome. Furthermore, depending on the origin of the EVs, they can potentially alter the phenotype of the growing cancer. Hypoxia-induced EVs, for example, contribute to a more aggressive and chemoresistant OvCa phenotype [20]. Briefly, OvCa cells exposed to hypoxic conditions significantly increased secretion of EVs that were carrying more potent oncogenic proteins, such as STAT3 and FAS, which can enhance metastasis/chemotherapy resistance in OvCa.

Considering this, EVs derived from the female reproductive tract may serve as therapeutic targets as well as biomarkers, since the identification of various factors, including immunosuppressive molecules, within their cargo has a huge therapeutic and diagnostic/prognostic potential for a variety of female reproductive tract-related disorders [21]. Currently, there are several databases available that gather information on the cargo that has been identified in the lumen and on the surface of EVs [22,23].

## 3. Extracellular Vesicles in Ovarian Cancer

Ovarian cancer (OvCa) is different from other solid tumours by its preference for peritoneal dissemination through ascites, which precedes hematogenic or lymphatic metastasis. Several studies have shown that EVs secreted by the cells from the tumor microenvironment (TME), whether by the tumor cells itself or by other cells like fibroblasts, mesothelial or immune cells, promote tumor survival and metastasis by many different mechanisms, including immunosuppression and immune evasion. It has been shown that EVs can either directly inhibit the anti-tumor function of diverse immune cells, e.g., by inducing apoptosis, suppressing T cell and NK-cell activation or inducing the expression of immune checkpoints on these cells, or indirectly by the recruitment of immunosuppressive cells such as regulatory T-cells (Treg) or myeloid-derived suppressor cells (MDSCs). EVs can also reprogram cells of the TME towards an immunosuppressive, pro-tumorigenic phenotype or reshape the TME metabolism in a way that it supports tumor growth. In the following section we describe these mechanisms in more detail divided by the type of cells being targeted.

### 3.1. EVs as Mediators of Immunosuppression in Ovarian Cancer

#### 3.1.1. EV-Mediated Suppression of T-Cells

Extracellular vesicles derived from sera of OvCa patients can directly inhibit CD8+ lymphocytes by inducing apoptosis through FasL present on their surface and the downregulation of CD3zeta expression [24]. Similarly, ascites-derived EVs have been shown to induce T-cell apoptosis and inhibit the expression of CD3zeta and Janus kinase 3 (JAK-3) [25]. Lysophosphatidic acid elevates the expression of FasL on the OvCa cell surface and induces the release of FasL-bearing EVs [26]. Another immunosuppressive molecule besides FasL found on EVs is GD3, a ganglioside expressed on ascites-EVs that causes arrest of a specific subset of T-cells, the so-called NK T-cells (NKTs). These cells share properties of both T-cells and NK-cells and constitute only 1% of all peripheral blood T-cells but can induce a very robust adaptive immune response. Many of them recognize the non-polymorphic CD1d molecule, an antigen-presenting molecule that binds self and foreign lipids and glycolipids. GD3 present on ascite-derived EVs compete with and displace the endogenous stimulatory ligands for CD1d molecules and thereby inhibit activation of NKTs by their natural stimulatory ligands [27]. Other researchers have found out that the inhibitory effect of GD3 is determined by the sialic acid group in EVs [28]. Another molecule that has been identified on small EVs (sEVs), plasma gelsolin (pGSN), has been already identified as a key factor of OvCa chemoresistance; however, recently, an additional immunomodulatory role of sEV-pGSN has been detected. Chemoresistant OvCa cells increase the secretion of sEV-pGSN, which induce CD8+ T-cell apoptosis and reduction of IFNγ production and further increase GSN expression in OvCa cells [29]. Similarly, phosphatidylserine (PS) carrying EVs derived from ascites reversibly inhibit T-cell activation by blocking NF-kβ- and NFAT-signaling pathways in T-cells [30]. 

Nucleic acids transferred by EVs may also suppress T cell functions. The circular RNA, circ-0001068, has been recently identified as a novel biomarker for OvCa. Circ-001068 was delivered by EVs to T-cells and induced PD-1 expression and T cell exhaustion by acting as a competing endogenous RNA for miR-28-5p [31]. Peng et al. co-incubated ascites-derived EVs with peripheral blood cells (PBMCs), dendritic cells (DCs) and hematopoietic stem cells and observed induction of apoptosis of these cells [32]. In summary, the data suggest that tumor-derived EVs convey inhibitory signals to T-cells by delivering ligands bound to the EV membrane to the cognate receptors on immune cells. Muller et al. observed that phenotypic and functional changes of T-cells upon EV interaction result in transcriptional alterations of multiple immunoregulatory genes [33]. Another group studied the effect of OvCa-derived EVs on the mRNA profile of T lymphocytes. They observed an up-regulation of 26 immunosuppressive genes, including IFNγCCL4/18, IL-10, Foxp3, IL-6 upon co-culture [34]. In further research, the group found t that ascite-derived EVs impaired T cell activation by up-regulating Singlec-10 on these cells, an inhibitory receptor from the sialic-acid binding immunoglobulin-type lectin group. Moreover, they detected that the expression of Singlec-10 and its ligand, CD24, in OvCa tumours was associated with advanced disease and cancer metastasis [35]. It was also found that EVs do not need internalization to impact T cell function [36]. Interestingly, the suppression of T-cells by OvCa-derived EVs can be reversed. The T cell inhibition observed by Shenoy et al., manifested as a blockade of nuclear translocation of NF-kβ and NFAT, downregulation of CD69, CD107a and cytokine production, as well as proliferation arrest, could be reversed within 24–48 h after the removal of EVs [37]. 

Additionally, OvCa-derived EVs are able to suppress T-cells indirectly by shaping TME metabolism. For example, tumor-derived EVs increase the production of extracellular adenosine. Adenosine is a well-known immunosuppressive factor and interacts via its receptors (A1, A2A, A2B and A3) expressed on various cell types, including lymphocytes. Signaling via the adenosine A2A receptor up-regulates cAMP levels in CD4+ effector T-cells, thereby reducing cellular functions [38]. Human natural Treg cells express CD39, but rarely CD73, although inducible Treg cells found in the blood and tumour tissues of cancer patients co-express both these enzymes [39]. Tumor-derived EVs, which are ubiquitously present in body fluids of cancer patients, carry CD73 and have 5′-nuclotidase activity that inhibits T cell activation through the adenosine A2A receptor. The functional ectonucleotidases CD39 and CD73 transferred by EVs, hydrolyse exogenous ATP and cAMP to adenosine [40]. EVs can also deliver CD73 to circulating CD39+CD73-Tregs to enable production of immunosuppressive adenosine [41]. Recently, another important metabolic checkpoint has been detected on OvCa EVs: arginase 1 (ARG1), one of the two isoforms of arginase. Arginases are important regulators of amino acid metabolism that affect the immune response and inflammation and are well known powerful immunosuppressants, especially in the tumor microenvironment (TME). It was shown that tumor cells can act through vesicular ARG1 beyond the TME, transferring functionally active ARG1 over long distances to the local lymph nodes, the key site of T cell activation and proliferation upon antigen recognition. There, the enzyme decreases the L-arginine levels, inhibiting T cell proliferation and therefore mitigating the anti-tumor immune response [42].

#### 3.1.2. EV-Mediated Suppression of NK-Cells and DCs 

NK-cells are the first line of defense within the TME and are important effectors in the cancer immune surveillance through the capability of direct tumor cell killing, so it is not surprising that tumor cells target via EVs NK-cells as well to evade the immune system. Tumor-derived EVs suppress NK-cells mainly by inducing downregulation of the NK surface receptors, particularly of NKG2D, a crucial mediator of NK cytotoxicity. Tumor cells, via EVs, secrete NKG2D ligands such as MHC class I chain-related proteins (MICA and B) or UL-16 binding proteins (ULBP) that upon interaction with NKG2D receptors on NK-cells surface determine their downregulation resulting in a general dysfunction of NK-cells [43]. Many studies have reported this EV-mediated effect for different types of cancer; however, reports concerning OvCa are rare. Labani-Motlagh et al. has shown that OvCa-EVs contain high levels of the KLRK1/natural killer group 2 (NKG2D) ligands in the manner of MHC I chain-related protein A and B and UL-16-binding protein. Their presence downregulates the expression of NKG2D receptors on NK-cell surface but does not affect another important cytotoxicity receptor, DNAM-1, suggesting that NKG2D might be a more prominent target for tumour cells [44]. Furthermore, it was shown that EV uptake by NK-cells is PS-dependent, since blocking with Annexin V prevented uptake by NKL cells [45]. However, since the authors also observed EV preparations that showed only minor uptake and were not affected by Annexin V despite PS-presence on the EV surface, they concluded that EV-associated PS alone is not sufficient for uptake, and other molecules such as lactadherin/MFG-E8 or integrins may be involved.

In the case of DCs, EV-DC interactions have been mainly described in the context of activation of immune responses and the usage of EVs as vaccines. However, multiple studies have also shown a negative impact of EVs on the differentiation, maturation and function of DCs in many cancer types, as reviewed in [46]. However, reports about OvCa are sparse. In the previously mentioned study about OvCa-derived ARG1+ EVs, it was shown that these vesicles not only directly inhibit T-cell proliferation but also act indirectly by impairing the activating potential of DCs in vitro and in vivo [42]. The ARG1+ EVs are readily internalized by murine bone marrow-derived DCs, which when co-incubated with CD8+ and CD4+ T-cells inhibited their unspecific and antigen-specific proliferation. The same EVs, applied subcutaneously, travelled to draining lymph nodes and inhibited OVA-specific proliferation of adaptively transferred OT-I T-cells, in contrast to recombinant ARG1. In all these described cases, pharmacological ARG1 inhibition partially reversed this EV-mediated suppression, providing a new target for immunotherapy approaches [42]. 

#### 3.1.3. EV-Mediated Suppression of Macrophages

Macrophages are, besides NK-cells, key effectors of innate immunity and are classically categorized into two polarized phenotypes that depend on their activation pathway: pro-inflammatory macrophages (M1) and anti-inflammatory macrophages (M2). Macrophages that permeate malignant tissues are called tumor-associated macrophages (TAMs) and are the main cells in the TME. In OvCa they usually manifest the M2-like phenotype and secrete IL-4, -5, -6 and -10 to promote angiogenesis, matrix remodeling and immune suppression. They foster an immunosuppressive microenvironment by secreting T cell modulating factors such as CCL-22, B7-H4, and CD206 [47]. OvCa-derived EVs usually drive the polarization of macrophages towards the M2 phenotype, often by the induction or transfer of specific miRNAs. Under hypoxic conditions, EVs secreted by OvCa cells activate hypoxia-inducible factors that upregulate the expression of miR-21-3p, -125b-5p and -181d-5p in macrophages and promote their polarization to the M2 phenotype through the (SOCS)4/5/STAT3 pathway [48]. Similar, hypoxia induces high expression of miR-940 in EVs from epithelial ovarian cancer (EOC) cells, which induces M2 polarization of macrophages [49]. miR-222-3p [50] or miR-1246 [51] transfer to macrophages induces M2-like polarization. MiR-222-3p targets the SOCS3 gene expression and induces STAT3 expression [50] in macrophages, and miR-1246 downregulates Cav1 in these cells, conferring chemoresistance [51]. EV-derived CD47, an integrin associated with inhibitory receptor interacting with SIPR-α, correlated negatively with macrophage infiltration and was linked to poor progression-free survival. Inhibition of EVs secretion and uptake enhanced phagocytosis of tumor cells by M1 macrophages and suppressed tumor progression in a xenograft mouse model [52].

### 3.2. Indirect EV-Mediated Immunosuppression

OvCa-derived EVs promote tumor immune evasion and tumor progression not only by direct inhibition of immune cells, but also indirectly by inducing and reprogramming of immune as well as non-immune cells in the TME, such as regulatory T-cells, fibroblasts, mesenchymal cells, and adipocytes. These activated/reshaped cells enhance or gain new immunosuppressive potential and, in turn, secrete their own EVs that exert this potential in the local TME, but also systemically.

#### 3.2.1. EVs Promote Differentiation, Expansion and Functions of Tregs

Regulatory T-cells negatively regulate the antitumor response and their elevated levels in the TME promote OvCa progression [53] and are correlated with the poor prognosis and shortened overall survival of OvCa patients [54]. Ascite-derived EVs, but not control EVs, induced Treg cell expansion in culture. These OvCa EVs effectively mediated conversion of conventional CD4+ CD25neg T-cells into CD4+ CD25highFOXP3+ Treg cells [55]. Interestingly, Treg cells were completely resistant to apoptosis mediated by tumor-derived EVs. Upon co-incubation with OvCa EVs, which were enriched in the membrane-form of TGFβ-1, Treg cells up-regulated expression of FasL, IL-10, TGFβ-1, CTLA-4, GrB and perforin, as well as of phosphorylated Smad2/3 and phosphorylated STAT3. All these changes increased their suppressive potential and apoptosis resistance. These EV-mediated effects were dependent on TGFβ-1 and IL-10, as neutralising antibodies specific for these cytokines blocked the ability of tumor-derived EVs to expand Treg cells [55]. 

#### 3.2.2. EV-Mediated Remodeling of Other Cells of the TME towards Immunosuppression

Myeloid-derived suppressor cells (MDSCs) are a heterogenous population of cells that are present in small numbers in a variety of tissues but expand during malignant transformation, inflammation and infection, and have a remarkable immunosuppressive capacity [56]. Accordingly, the presence of MDSCs in cancer patients is associated with poor survival and tumor progression and is considered a major limitation for cancer immunotherapy [57]. It was shown that tumor-derived EVs can activate MDSCs through membrane-anchored HSP70. EV-associated HSP70 binds to the toll-like receptor 2 (TLR2) that leads to the stimulation of the nuclear factor–kappa B signaling pathway and then activation of the signaling pathway Janus kinase 2 (JAK2)/STAT3 through IL-6 autocrine secretion [58]. HSP70 has been detected on the surface of EVs derived from urine of OvCa patients that could be captured by the HSP70-specific peptide aptamer, A8, targeting the extracellular domain of HSP70. The number of HSP70 EVs was higher in all cancer patients compared with healthy donors, for whom hardly any HSP70 EVs could be detected, although the total EV number was higher [59]. Once activated, the MDSCs in turn start to produce their own EVs, which also have immunosuppressive properties, further driving the tumor immune evasion process [60]. 

Fibroblasts are another type of non-tumor cell in the TME that are targeted and remodelled by tumor-derived EVs to sustain tumor immune escape, and are an important component of the tumor stroma, which accounts for a large percentage of the tumor tissue in OvCa. Tumor-conditioned fibroblasts have been proven to contribute in large part to OvCa progression [61]. OvCa cell line-derived EVs have been shown to activate normal fibroblasts to a CAF-like state with a higher rate of proliferation, motility, invasiveness, enzyme expression and, interestingly, EV release. These fibroblasts expressed the common markers for CAFs, including α-SMA, SDF-1, FSP-1, vimentin, desmin, tenascin and FAPα. The secretome of these “activated” fibroblasts including EVs was, in turn, able to modulate the responses (proliferation, motility and invasion) of other fibroblasts, as well as tumor and endothelial cells. EVs released from a more aggressive cell line (SKOV3), seemed to be more efficient in the CAF stimulation compared with EVs released from a less aggressive cell line (CABA I). EV-associated TGFβ could be one of the molecules responsible for these processes. The authors assume that TGFβ may take part in this EV-mediated fibroblast activation [62]. Similar, EVs secreted by epithelial OvCa induced adipose tissue-derived mesenchymal stem cells to differentiate into tumor-associated myofibroblasts and upregulate tumor-promoting and immunosuppressive factors such as stromal cell-derived factor and TGFβ [63]. Similar to MDSCs, CAFs secrete EVs with tumor-promoting potential, e.g., by transfer of TGFβ-1 and SMAD signaling activation driving EMT [64].

### 3.3. Therapeutic Applications of EVs in Ovarian Cancer

#### 3.3.1. EVs as Drug Carriers 

Recent advances in the translational research in OvCa have demonstrated that EVs can serve both as efficient therapeutic modalities, e.g., as drug carriers or vaccines, as well as therapeutic targets, as reviewed in [65]. Due to their high biocompatibility, enhanced stability and limited immunogenicity, EVs are, for example, ideal drug carriers and provide multiple advantages in comparison to synthetic nanoparticles. Studies have shown that natural EVs are preferentially taken up by cancer cells in comparison to normal cells and, based on their specific tetraspanin and integrin cargo, can target selected cells. They have been used for the delivery of small molecule drugs, miRNAs, proteins and siRNAs in preclinical studies of various cancer types, as reviewed in [66]. In the case of OvCa, EV-based delivery of triptolide (TP), an active herbal substance with proven anti-tumor, immunomodulatory and anti-inflammatory potential, showed enhanced anti-cancer effects and limited toxicity in comparison to free TP. TP-EVs induced OvCa-cell apoptosis and inhibited tumorigenic M2 macrophages [67]. Although possessing promising potential, EV-based drug delivery remains challenging due to lack of standardised isolation and purification methods, limited drug loading efficiency, and insufficient clinical grade production. As a possible solution, the development of so-called Immune Derived Exosome Mimetics (IDEM), providing the desired scalability and bioavailability, has been proposed [68]. These IDEMs have been shown to be more efficient in the delivery of therapeutic active doxorubicin to OvCa cells than the free drug, allowing reduction of the drug dose to reduce side effects. Additionally, numerous studies have revealed that also miRNAs or siRNAs are carried by EVs and function either as oncogenes or tumor suppressors in EOC. Their role in the progression and chemoresistance of OvCa, their significance for therapy and as non-invasive biomarkers, has been widely studied and has been comprehensively reviewed elsewhere [69,70].

#### 3.3.2. EVs as Cancer Vaccines

Dendritic cell-derived EVs have been shown to carry peptide-MHC I and -MHC II complexes on their surface, even at a higher amounts than DCs themselves. The proven cross-presentation of these EV-bound antigens was shown to be particularly efficient because of the presence of antigens in the unique environment of the tetraspanin protein-enriched membranes of EVs. Having higher stability, being more cost- and time-effective and, most importantly, being less prone to immunosuppressive mechanisms in the TME than DCs, they have emerged as a new option for cancer vaccination. Showing a high immune-activating potential in pre-clinical studies, they have already entered clinical trials. First phase I trials of DC-derived EVs were conducted in lung cancer, melanoma and colorectal carcinoma, and promising antigen-specific T cell responses were observed, although in the most studies significant therapeutic effects were only observed in combination with other immunostimulatory agents [71,72,73,74]. First steps towards EV-based immunotherapy of OvCa have also been undertaken, though only at a pre-clinical level so far. A method for the production and purification of clinical GMP-grade EVs from ascites of OvCa patients has been developed [75] for use in a future clinical trial in conjunction with the double-stranded RNA adjuvant polyI:polyC12U (Ampligen^®^) [76]. Interestingly, it was shown that EVs from OvCa ascites efficiently translocate mucin 1 (MUC1) from the endolysosomal/HLA-II to the HLA-I compartment, accompanied by deglycosylation that generates novel MUC1 moieties that are presented more effectively by DCs to MUC1-specific CD8+T-cells [77].

Additionally, numerous studies have provided evidence that tumor-derived EVs carry many different tumour antigens that can induce cellular and humoral immune responses in cancer-bearing patients. EV-associated tumor antigens such as HER2, EPCAM or Melan-A/MART-1 have been detected in the circulation and/or other body fluids in several types of cancer, including OvCa [78] and, accordingly, HER2- or EpCAM-specific antibodies are detectable in the sera of patients with HER2+ and EpCAM+ cancer, implementing a humoral immune response [79,80,81]. Based on these findings, the mentioned antigens have been used as targets for cancer immunotherapy. A number of therapeutic monoclonal antibodies and their derivatives against these antigens have been developed to target them on the tumor-cell surface to elicit, e.g., antibody-dependent cellular cytotoxicity (ADCC). In this light, the release of immunogenic vesicles by tumour cells seems at first glance paradoxical, since tumours aim, as we have shown before on multiple examples, towards immune evasion. Battke et al. provided a possible explanation for this obvious contradiction. The authors show that EVs isolated from BrCa cell lines or OvCa ascites bound and sequestered tumour-reactive antibodies, such as the therapeutical anti-HER2 monoclonal antibody trastuzumab and an anti-EPCAM tumor-reactive antibody. This resulted in a reduced binding of these antibodies to the tumour cells and, as a consequence, impaired antibody-dependent cellular cytotoxicity (ADCC), which is a major anti-tumor immune effector function [82]. A very similar effect was observed in the case of EVs released by highly aggressive B-cell lymphoma cell lines that protect the tumor cells from rituximab-mediated cytolysis by intercepting the mAbs through CD20 present on their surface [83]. Therefore, these EV-mediated decoy effects may be a critical determinant of tumor cell susceptibility to immunotherapy and be responsible for the often observed resistance of patients to mAb therapy. Lowering the levels of EVs carrying antibody targets might improve immunotherapy efficacy or sensitise patients to mAb-based therapies. An alternative approach could be to capture such EVs with macrophages or DCs, or by using scaffolds functionalized with EV-binding molecules. In this manner a pre-metastatic niche mimic, named M-trap, was generated by embedding OvCa EVs into biomaterial scaffolds and implanted within the abdominal cavity of mice. This artificial pre-metastatic niche effectively trapped OvCa tumor cells from the ascites, preventing metastasis and increasing survival [84]. 

#### 3.3.3. EVs as Therapeutic Targets

Since the cargo of tumor-derived EVs promotes tumor immune evasion by different mechanisms, blockage of EV production, secretion, cell-targeting, and signaling have been recently developed as potential anti-cancer therapies. Studies on drugs inhibiting EV biogenesis and secretion at different levels are still in their pre-clinical stage, including the blockage of the ESCRT-dependent or the ESCRT-independent mechanism-mediated EV biogenesis, or the inhibition of syndecan/syntenin/ALIX, sphingomyelinases, Rab-proteins and small GTPases (reviewed in [85]). Substantial efforts have still to be made to investigate the concentration-response effects of these compounds on vesicle release, their off-target effects and their impact on healthy cells. Especially in the case of cancer, the development of approaches for selective drug delivery only to cancer cells is important. Most drug inhibitors have only been tested in vitro and in pre-clinical mouse models (in [86]). It is already known that the cell- and organo-tropism of EVs are dependent on ligand-receptor interactions on the surface of EVs and target cells. Hoshino et al. demonstrated that distinct surface integrin patterns on EVs can determine preferential targeting of specific organs such as liver or lungs, providing a first rationale for the development of EV integrin-blocking strategies; for instance, with decoy peptides, to prevent organ-specific metastasis [87]. 

The development of immune checkpoint blockade (ICB) therapies, including PD-1/PD-L1 and CTLA-4 inhibitors, in the last decade has been a breakthrough in immuno-oncology [88]. Seven monoclonal antibodies targeting the PD-1 (pembrolizumab, nivolumab, and cemiplimab), PD-L1 (atezolizumab, durvalumab, and avelumab) and CTLA-4 (ipilimumab) have been approved for the treatment of different malignancies such as melanoma and NSCLC, and showed impressive long-term results, but only in subgroups of patients. Recently, PD-L1 and other immune checkpoints have been identified on the surface of tumor-derived EVs in melanoma, head-and-neck cancer, NSCLC and glioblastoma (reviewed in [89]). These discoveries provide new insights on the mechanism by which tumor cells suppress the immune response as well as provide a possible explanation for the low response rates of ICB therapies. Preclinical and clinical studies indicated not only that EV-associated PD-L1 strongly contributes to immune suppression, but also can reduce the efficacy of PD-L1 blocking therapy, possibly by acting as decoy for anti-PD-L1 antibodies in a similar way described above for trastuzumab and rituximab (reviewed in [90]). Poggio et al. showed that removal of EV-associated PD-L1 can increases the response rate of the anti-PD-L1 blockade and induce long-term systemic immunity to PD-L1-positive tumours, supporting the critical impact of EV-associated PD-L1 in therapeutic resistance to anti-PD-L1 antibody treatment [91]. Thus, the effectiveness of immune checkpoint therapies might be increased in combination with strategies reducing EV-associated PD-L1 levels. Furthermore, EV-bound PD-L1 has emerged as a new predictive biomarker of response to ICB. Indeed, it was shown in melanoma that the levels of circulating EV-PD-L1 (but not soluble PD-L1) could differentiate responders from non-responders [92]. Regarding OvCa, although at least a third of studied OvCa tumours were positive for PD-L1, its clinical impact was not clearly elucidated, with conflicting results regarding the association with higher tumor stage/grade or shorter survival. So far, no ICB therapy has been approved for OvCa. Until now, several clinical studies targeting PD-1, PD-L1 and CTLA-4 have been undertaken, but there has been no reported improvement in survival, and some trials were terminated early due to toxicity or lack of response (reviewed in [93]). High levels of PD-L1-EVs in OvCa might be an explanation for this low efficacy but, surprisingly, the presence of PD-L1 EVs or EVs carrying other immune check-points in OvCa has not been confirmed so far, leaving space for further discoveries in the future.

While counteracting tumor EV-decoy function might improve immunotherapy efficacy, as described above, the same EV property might be advantageous when used therapeutically to neutralize the activity of inflammatory molecules such as IL-6 or TNFα. Zhang et al. combined both the decoy and the drug delivery properties of EVs to use them therapeutically. The authors engineered EVs displaying PD-1 on their surface, which, by shielding PD-L1 on melanoma cancer cells, disrupted the immunosuppressive PD-1/PD-L1 axis. At the same time, the authors loaded into these modified PD-1+ EVs an inhibitor of indoleamine 2,3-dioxygenase (IDO) to block a parallel immunosuppressive pathway, which allowed increased tumor infiltration of CD8 + T-cells and tumor regression [94].

## 4. Extracellular Vesicles in Endometrial Cancer

Endometrial carcinoma (EC) is one of the most common gynecological cancers. Most cases are diagnosed in the early stage, which assure a 5-year survival rate of over 95% [95]. However, cases of recurrent or metastatic EC are linked to poor prognosis [96,97]. EC has been classified into two main types: type I and type II. Type I is an endometrioid adenocarcinoma that is considered to have a more favorable outcome, whereas type II includes several subtypes such as serous, clear cell, or undifferentiated carcinomas that are linked to poorer survival rate [95]. Emerging evidence suggests that endometrial cancer may progress due to immunological impairment in the TME [98]. Recent studies showed that EVs may play a role in this process.

### 4.1. EVs as Mediators of Immunosuppression in Endometrial Cancer

#### 4.1.1. EV-Mediated Suppression of Macrophages

Li Xiao et al. demonstrated that EC cells under hypoxic conditions released higher amounts of EVs than in normal conditions. Moreover, EC-derived EVs contained a high amount of miR-21 that was transported to the monocytes. This EVs-derived miR-21 induced an expression of IL-10 and CD206 in THP-1 cells, which suggests the polarization of monocytes into the M2 phenotype [99]. These results indicate that EVs-derived miR-21 may play an immunosuppressor role by modulating monocyte polarization in the EC microenvironment. This finding may be supported by the work of other researchers from Zhengzhou University. Gu et al. showed that EC specimens contained a higher amount of M2 macrophages than normal endometrial specimens [100]. The flow cytometric quantification showed that tumor-associated macrophages obtained from EC specimens expressed more CD206 than those derived from healthy specimens. Furthermore, scientists observed a higher amount of M2-associated mRNAs for e.g., IL-10, Arg-1, and TGFβ and the reduction of M1-associated mRNAs such as TNF-α, IL-6, and IL-12 in tumor-associated macrophages from EC specimens, in comparison to macrophages obtained from healthy specimens [100]. 

#### 4.1.2. EV-Mediated Immunosuppression of Other Non-Immune Cells

Higher levels of M2 macrophages in the TME of EC may act not only by suppressing the immunological response but may also take part in enhancement of tumor viability and resistance. Gu et al. showed that M2-macrophages produced EVs that were taken up by EC cells, which resulted in decreased radiosensitivity of the tumor. Further investigation revealed the potential mechanism behind this phenomenon. M2 macrophage-derived EVs contained a high amount of hsa_circ_0001610 that was transported to the EC cells where it suppressed miR-139-5p expression. Moreover, downregulation of miR-139-5p resulted in higher expression of cyclin B1, which is a crucial enhancer of radio-resistance [100].

### 4.2. Therapeutic Applications of EVs in Endometrial Cancer

Research suggests that EVs play a role in EC progression by modulating macrophage polarization [99]. The EVs responsible for the effect contained a high amount of miR-21, which is an interesting candidate for the development of therapeutic strategies. Moreover, EVs excreted by macrophages contributed to enhanced EC radio-resistance by transporting hsa_circ_0001610 to the EC cells [100]. This evidence suggests that EV-associated hsa_circ_0001610 may be considered another interesting therapeutic target. The potential of blocking these two EVs components in EC is promising. Unfortunately, to date there are no registered clinical trials with compounds that would act as EVs miR-21 or EVs hsa_circ_0001610 inhibitors in patients with EC. This type of strategy may become a significant tool in treatment against EC, especially type II which is hard to treat and has a poor survival rate. Interestingly, to date there is an ongoing clinical trial focused on caloric restriction before surgery in patients with EC and its effect on miR-21 serum level [101]. The outcome of this study may increase interest in the field and convince scientists to further investigate the EVs miR-21 blockage effect in EC patients. Another potential therapeutic strategy may be the inhibition of EV production by EC cells. Established compounds with such effects include calpeptin, manumycin A, imipramine, and many more [85]. Some of those compounds are registered for treatment in other human diseases. Imipramine for instance is a well-established drug for depression and anxiety treatment, that has reported side-effects such as dizziness, sedation, constipation, dry mouth, urinary retention, weight gain, confusion, or even arrhythmia [102,103]. However, the side effects of other compounds, which are not commonly used and not registered in human treatment, have to be further investigated. Since EV secretion is a physiological process typical for all cells, including healthy cells, this type of therapeutic strategy may be not selective and may provoke many side-effects [104]. 

## 5. Epidemiologic and Genetic Connections between Ovarian Cancer, Endometrial Cancer and Endometriosis

Endometriosis is a relatively common, benign gynecological disorder with chronic inflammatory characteristics [105]. It has been shown that endometriosis, the etiology and epidemiology of which is described in detail in the next section, is linked to oncological pathologies such as OvCa [106]. The pooled analysis of case-control studies conducted by Pearce et al. revealed that 9.3% of women with invasive OvCa and 6.2% with borderline OvCa had a history of endometriosis [107]. The incidence of endometriosis history in patients with OvCa varied depending on the histological type of the malignancy. A total of 20.2% of women with clear-cell ovarian carcinoma reported an endometriosis history. Endometriosis was also reported by 13.9% of women with endometrioid ovarian carcinoma, 6.0% with mucinous, 7.1% with high-grade serous, and 9.2% with low-grade serous type. Among borderline OvCa, 9% of women with borderline serous type and 8.5% with borderline mucinous type reported endometriosis history [107]. Endometrioid and clear cell ovarian cancers have the strongest correlation with endometriosis among all endometriosis-associated ovarian cancers [108]. Gene alterations found in both endometriosis and clear-cell ovarian carcinoma include genes such as PIK3CA, PTEN, ERBB2, KRAS, ARID1A, PPP2R1A, MLH1, and CTNNB1. Furthermore, ovarian endometrioid cancer shares similar gene alterations with endometriosis, including PIK3CA, PTEN, KRAS, and ARID1A [108].

Endometrial cancer’s association with endometriosis is debatable. Many studies confirmed no significant correlation between endometrial cancer and endometriosis [109,110,111,112]. Conversely, Mogensen et al. demonstrated a significant correlation between endometriosis and Type I endometrial cancer [113]. Genetical analysis has shed a little more light on the subject. Alteration in the PTRD gene was found in both endometriosis and endometrial cancer [114]. Other potential candidates for gene alterations correlated with both endometriosis and endometrial cancer are located nearby the SKAP1 and DUSP6 genes. However, gene alterations in PTRD as well as in SKAP1 and DUSP6 regions require further experimental studies to validate their actual contribution to the development of endometriosis and endometrial cancer [114]. Despite their potential similar genetic origin, more studies are needed to decide whether endometriosis is connected to endometrial cancer on a clinical and molecular basis.

## 6. Extracellular Vesicles in Endometriosis

Endometriosis is a chronic inflammatory gynecological disorder coupled with infertility and pelvic pain. It is characterised by estrogen-dependent growth of endometrial and stromal tissue at aberrant locations outside the uterus [105]. It affects roughly up to 10–15% of women of reproductive age [115], which extrapolates to around 190 million women worldwide [105]. Despite its high incidence, the origin of endometriosis remains unknown, with a lack of definitive biomarkers, and necessitating surgical intervention for diagnosis [116]. This creates an urgent need for the development of innovative non-invasive diagnostic techniques as well as the discovery of effective therapy targets in order to enhance clinical outcomes for patients. Further study is needed to better understand the fundamental pathophysiological pathways that facilitate endometriosis development.

One of the most widely accepted theories regarding endometriosis development is Sampson’s theory of retrograde menstruation [2,117]. Briefly, it holds that endometriosis arises from the implantation of sloughed endometrial tissue refluxed into the pelvis via the fallopian tube(s) during the menstruation period [2,117]. Although the phenomenon of retrograde menstruation is observed in most menstruating women, it remains unclear why only some of them develop this pathological condition. A possible explanation for this is a dysfunction of the immune system in terms of defective immune surveillance against autologous tissue deposited in the peritoneal cavity [118]. Indeed, recent studies have drawn links between the aetiology of endometriosis and immune dysregulation in women with this disease [116]. Studies show that almost all types of immune cell present in peripheral blood and peritoneal fluid of endometriosis patients are more or less dysfunctional, creating a complex immune milieu favouring disease progression, as reviewed in [119,120]. As a result of the weakened immune response, ectopic endometrium cannot be cleared from the pelvic cavity, allowing it to escape immune monitoring and implant and grow outside the uterus, resulting in formation of endometriotic lesions and disease progression [121].

**Figure 2:**
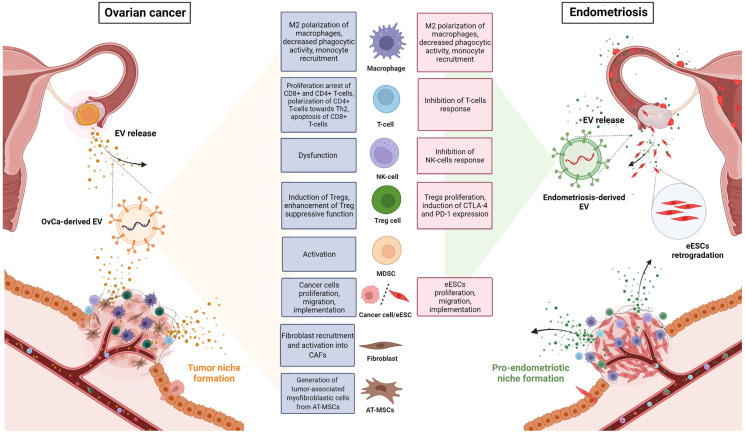
Comparison of extracellular vesicle (EV)-mediated effects on diverse immune and non-immune cell types in tumor and pro-endometriotic microenvironments that promote tumor or endometriotic niche formation. Abbreviations: AT-MSCs, adipose tissue-derived mesenchymal stem cells; CAFs, cancer-associated fibroblasts; CD8+ T-cells; cytotoxic T lymphocytes; CTLA-4, cytotoxic T lymphocyte antigen 4; eESCs, ectopic endometrial stromal cells; M2, pro-inflammatory phenotype; MDSCs, myeloid-derived suppressor cells; MSCs, mesenchymal stem cells; NK-cells, natural killer cells; OvCa, ovarian cancer; PD-1, programmed cell death protein 1; T-cells, T lymphocytes; Th2, T helper cell type 2; Tregs, regulatory T lymphocytes. This image was created using BioRender (http://biorender.com/ accessed on 22 April 2022).

Endometriosis is considered a benign gynecological disorder, but it has distinctive biological behaviours that mimic those of malignant tumours, particularly OvCa [122], such as tissue invasion, immune dysfunction, spread to distant organs, vascularisation and innervation of endometriosis lesions [122] (Figure 1). As with OvCa, the dynamic process of endometriosis progression is related to changes in the microenvironment in the peritoneal cavity, as reviewed in [120]. An important role in both pathologies is largely determined by the existence of a supportive and receptive “niche microenvironment” inhabited by a variety of stromal and immune cells [123]. Extracellular vesicles in endometriosis, as in OvCa, are thought to play a key role in disease development as intracellular communication mediators, as reviewed in [117] (Figure 2). An increasing number of studies have shown that EVs secreted by cells from the peritoneal cavity, whether by immune cells themselves or released from endometriotic lesions, act as immunosuppressive mediators, dysregulate immune system function by many different mechanisms, and facilitate the establishment of a chronic inflammatory and immunosuppressed microenvironment, termed the “pro-endometriotic niche”, that is favorable for the advanced progression of initial lesions, as reviewed in [120,124]. 

Exploring the potential involvement of endometriosis-derived EVs in endometriosis progression, and the influence they may have on immunological dysfunction during disease progression, is of great interest, considering the urgent need for innovative diagnostic and treatment strategies. In the following section, we summarize the current knowledge of the role of EVs as immunosuppressive variables in the chronic pro-inflammatory milieu of endometriosis and try to characterise these mechanisms in further depth.

### 6.1. EVs as Mediators of Immunosuppression in Endometriosis

Although the significance of EVs in tumor-induced immunosuppression has been extensively explored, less is known about their involvement in immune dysfunction in chronic inflammatory disorders such as endometriosis. 

The cargo of EVs isolated from the blood, peritoneal fluid, or endometriomas of women with endometriosis varies significantly from controls [125,126,127,128], indicating that EVs play an important role in disease progression, as reviewed in [10]. A few studies have recently shown that EVs transport several factors that modulate the development of endometriotic lesions, as reviewed in [117]. Through their proteomic and genetic (lncRNA, miRNA) cargo they may drive inflammation, as well as attenuate immune response [128,129], promote angiogenesis [130] and increase proliferation and motility of endometrial stromal cells (ESCs) [131,132]. 

The immunosuppressive character of EVs associated with endometriosis, which is the subject of this theoretical elaboration, has been attributed in a large part to their nucleic acid content, which is a key regulator of immune cell development and function. Various reports have indicated that an analysis of non-coding RNAs (ncRNAs), including miRNA and lncRNA, in both endometriosis and control EVs, shows that EVs may mediate distinct immune suppressive functions [128,129,131,132]. A recent study by Khalaj et al. demonstrated the presence of unique signatures of miRNAs and lncRNAs in the cargo of EVs isolated from women with endometriosis compared to controls [128]. Dysregulated transcripts were enriched in the signaling pathway target genes related to endometriosis, as well as with immune and metabolic functions [128]. Their results revealed that endometriosis-associated EVs carry a distinctive cargo that influences disease progression through the modulation of inflammation, angiogenesis, and proliferation [128]. Furthermore, Chen et al. discovered a complex relationship between EV miRNAs and inflammation in endometriosis patients [129]. Extracellular vesicles isolated from endometriosis patients included 173 miRNAs that were differentially expressed when compared to the miRNAs in EVs of the control group [129]. MiRNA expression also varied depending on endometriosis stage. Thirteen miRNAs considered as mediators of inflammation and T cell immune response, including as miR-451a, were differently expressed in women with moderate to severe stages compared the control group [129]. Moreover, EV miRNAs associated with alteration of Treg and macrophage functions, such as miR-451a, miR-1908, and miR-130b, were also found in endometriosis patients, although the stage of disease was not determined in this case [129].

#### 6.1.1. EV-Mediated Suppression of Macrophages

Macrophages are the most abundant immune cells in endometriosis lesions and, together with NK-cells, are key players in the pathogenesis of endometriosis. Being highly plastic effector cells, macrophages can polarize toward one of two phenotypes, the pro-inflammatory (M1) or anti-inflammatory (M2) phenotype, depending on the milieu conditions. Multiple reports have demonstrated an aberrant distribution of macrophages inside the peritoneal cavity of endometriosis patients [133]. An abnormal hypoxic endometriotic milieu, in particular, can stimulate M2 polarization of macrophages, promoting ectopic lesion development and vascularisation via wound healing and tissue remodeling properties [134,135]. In endometriosis, the interplay between macrophages and ectopic endometrial cells is mainly regulated by EVs, as reviewed in [10]. It was revealed that EVs secreted by ectopic endometrial lesions interact with macrophages leading to their alternative activation. These reshaped macrophages gain immunosuppressive properties contributing further to the progression of endometriotic lesions in the peritoneal cavity. 

In the mouse model of endometriosis, Sun et al. demonstrated that EVs released from endometriotic lesions preferentially polarized macrophages towards an M2 phenotype with decreased phagocytic activity compared to macrophages from the control group, both in in vitro and in vivo experiments [134,136]. Actually, the volume and weight of ectopic lesions increased together with the number of infiltrating M2 macrophages, accelerating the progression of endometriosis lesions in mice. Macrophage recruitment was supported by EVs, similar to previously reported findings of Bacci et al. [134]. The mechanism by which endometriosis-derived EVs systemically affect macrophage function may be dependent on the action of miRNAs, which can influence genes in distant cells when transported as a part of the EV cargo. In the study of Huang et al., the endometriosis-derived EVs were reported to stimulate M2 phenotypic polarization of macrophages by the transfer of miR-301a-3p, which activated the PTEN/PI3K signaling pathway by up-regulating PI3K expression and down-regulating PTEN expression [137]. This effect was restored by addition of a miR-301a-3p inhibitor [137].

Extracellular vesicles are known to be produced and released in large numbers in inflammatory states [138]; therefore, macrophage-derived EVs are recognized as key mediators of the inflammatory response in endometriosis [139]. The escalation of inflammation in the peritoneal niche leads to the recruitment of naive monocytes from peripheral circulation to the inflamed tissue, where they have been shown to differentiate upon the uptake of EVs released by activated macrophages [139].

It was discovered that macrophage-derived EVs contain high levels of functionally active miR-223, which is the most abundant miRNA dysregulated in macrophage-derived EVs in endometriosis patients [140]. This miR-223 was shuttled to target cells via EVs where it stimulated differentiation of recipient monocyte, but was also taken up by endothelial, epithelial, and fibroblast cells [139]. Furthermore, it was observed that during the macrophage differentiation process, the monocytic cells selectively releases miR-223 to permit completion of the maturation process [139]. As a result, the EV-associated miR-223 can be shuttled to other progenitor lineages to complete their terminal maturation into granulocytes or megakaryocytes [139]. Therefore, M2-derived EVs containing high levels of miR-223 were found to activate hematopoietic cell production in the bone marrow and to induce the release of more EVs [139]. 

In conclusion, the EVs released by ectopic endometrial lesions interact with macrophages, resulting in their alternative activation. As a consequence, these altered macrophages gain new immunosuppressive potential, resulting in decreased phagocytosis and antigen-presenting abilities, which leads to defective immune clearance of the sloughed ectopic endometrial tissue in the pelvic cavity. Additionally, these remodelled macrophages release their own EVs, which exhibit their immunosuppressive potential, not only in the local peritoneal milieu, but also systemically—acting on both immune and non-immune cells. Overall, the accumulation of these compromised macrophages in the pelvic cavity may enhance the survival, proliferation, and implantation of ESCs, thus supporting the formation of an immunosuppressive pro-endometriotic niche to support further progression of endometriosis [141]. 

#### 6.1.2. EV-Mediated Suppression of T-Cells

Endometriosis-derived EVs can suppress T-cells indirectly by shaping the pro-endometriotic niche metabolism. For example, miR-301a-3p overexpression in vitro, as well as in vivo experiments involving endometriosis-derived EVs administration, significantly increased ARG1 expression in macrophages [137]. As discussed above in the section about OvCa, ARG1 is a powerful immunosuppressive enzyme that, when activated, causes local depletion of L-arginine, a necessary amino acid for effective T cell function. As a result, ARG1+ macrophages may suppress T cell activation in a similar manner in endometriosis to that in tumors [142], affecting the immunological response and deriving inflammation in the pro-endometriotic niche. However, in contrast to OvCa-derived EVs that have already been demonstrated to carry ARG1 [42], so far, no studies have confirmed the presence of ARG1 in the cargo of endometriosis-derived EVs. 

In another study, Texidó et al. demonstrated the presence of the ectonucleotidases CD39 and CD73 in the cargo of EVs isolated from ovarian endometrioma aspirates [143]. These ectonucleotidases-containing EVs were found to suppress local immune responses by modulating extracellular ATP and increasing extracellular levels of adenosine [143]. A similar mechanism was observed for OvCa-derived EVs, as described in more detail above. Extracellular adenosine, which is produced by the ectonucleotidases CD39 and CD73, is a newly identified “immune checkpoint mediator”. It acts as a potent immunomodulator, facilitating immunosuppression by inhibiting T and NK-cell responses and promoting Treg proliferation, CTLA-4, and PD-1 expression [144]. 

#### 6.1.3. EV-Mediated Immunosuppression of Other Non-Immune Cells

Endometriosis-derived EVs have been demonstrated to induce immunological dysfunction not only by directly inhibiting immune cells, but also by shifting non-immune cells in the pre-endometriotic niche towards an immunosuppressive phenotype. The secretome of alternatively activated macrophages, including EVs, has been shown to modulate ectopic endometrial stromal cells (eESCs), contributing to their proliferation, motility, and invasion, and thus promoting the establishment of endometriotic lesions [131,132]. For instance, in a cell proliferation and wound healing experiment, Zhang et al. noticed a significant increase of proliferation and migration of ectopic endometrial eESCs following exposure to EVs from peritoneal macrophages [132]. This effect was thought to be mediated by the EV cargo containing high levels of miR-22-3p, a cell proliferation regulator, and aimed at targeting the SIRT1 and activating the NF-κβ pathway [132].

Furthermore, in endometriosis the cargo of EVs from peritoneal macrophages contained a notably increased level of lncRNA CHL1-A, which acts as a competing endogenous RNA of miR 610 to induce MDM2 expression [131]. Liu et al. observed that this lncRNA appears to be transferred from macrophages to ectopic endometrial stromal cells via EVs, and promotes the proliferation, migration, and invasion of eESCs while blocking their apoptosis by downregulating miR-610 and upregulating MDM2 [131]. By these means, this EV-based on bi-directional communication between macrophages and ectopic endometrium drives the vicious cycle of endometriosis progression.

### 6.2. Therapeutic Applications of EVs in Endometriosis

Although endometriosis affects many women across the world, at present there are no effective therapies to either cure or provide reemission of endometriosis clinical manifestations. Due to the lack of available tools to diagnose or treat patients in the early stages, surgery is regarded as the only treatment for advanced cases. An early detection of endometriosis is critical to improve clinical outcomes for patients and, thus, there is an urgent need to better understand the molecular process behind disease development, as well as to find reliable non-invasive diagnostic biomarkers and effective therapy targets.

Since EVs are stable in blood circulation, have low immunogenicity, and act as carriers for functionally active biological molecules, they have great potential as diagnostic and/or prognostic biomarkers and therapeutic targets in endometriosis [145]. However, further research is required to develop methodologies for isolating distinct subpopulations of EVs and analysing their contents in a simple and precise manner before they can be used in clinical trials [146].

Various endometriosis-related genetic biomarkers have been identified through sequencing of the EV-cargo [125,126,147]. These novel molecules found within EVs from stromal cells, peritoneal fluid [147], and serum [125] of endometriosis patients, comprise lncRNAs, miRNAs, and mRNAs and may serve as diagnostics markers with prognostic value, as well as molecular targets for endometriosis therapy.

Possible therapeutic roles of EVs in endometriosis, apart from their diagnostic/prognostic potential, have been explored only in a few studies [148,149]. For instance, one of the main clinical issues associated with endometriosis is the accumulation of fibrotic tissue [150,151], which causes pelvic pain and infertility [115]. Wu et al. observed that increased fibrosis in endometriosis patients correlated with elevated expression of the fibrotic markers collagen αI, αSMA and CTGF [149]. The expression of miR-214, a miRNA with fibrosis-suppressing effects [152], was decreased in ectopic endometrium compared to normal endometrium [149]. However, delivery of miR-214 via EVs decreased the levels of mRNAs for CTGF and collagen αI in eESCs and epithelial cells in in vitro experiments [149]. Moreover, injections of miR-214-enriched EVs in a mouse endometriosis model resulted in a decreased score of fibrotic markers such as collagen αI and CTGF [149]. Therefore, the authors suggest that the EV-mediated delivery of miR-214 to eESCs may, in the future, be developed as a non-invasive gene therapy for endometrial fibrosis [149].

Recently, researchers have started to focus on the therapeutic application of EVs to restore endometrial function and minimize scar formation associated with fibrosis, including the use of therapies involving stem cell-derived EVs [153]. Stem cell therapy has attracted a lot of attention in the field of treating reproductive disorders [148,154,155], but the use of stem cell-derived EVs appears to be a novel therapeutic approach. It retains and even adds, to the benefits of stem cell therapy, such as safety, non-toxic properties, stability, and the ability to easily cross capillaries and the blood-brain barrier, as well as the potential for large-scale production, while avoiding stem cell therapy’s disadvantages, such as risk of teratogenesis [156]. According to data, mesenchymal stem cells (MSCs) are cells that produce the most EVs [157]. It was observed that EVs derived from human umbilical cord MSCs (UC-MSCs) can efficiently suppress ESC proliferation and invasion, as well as their expression of SF-1, ER, and aromatase, potentially leading to endometriosis alleviation [148]. As a result, when compared to the previous studies, stem cell-derived EVs show more promising potential as a non-cellular treatment to combat endometriosis and should be explored as a new therapy for this chronic inflammatory disorder in the future.

Better understanding of the EV-mediated mechanisms that drive the establishment of the pro-endometriotic niche may inspire novel EVs-based strategies for endometriosis early diagnosis and therapy. Blocking of the molecular components delivered via EVs from the endometriotic lesion, blocking the EV-mediated recruitment and activity of immunosuppressive cells, and constricting the EV-driven angiogenesis process, represent only few potential EVs-based strategies for preventing endometriosis progression. Future studies should examine the composition of endometriosis-derived EVs as well as develop models to assess the mechanisms driving aberrant EV synthesis. Significantly, studies examining whether tailored therapeutic approaches might attenuate EV content and limit establishment of ectopic lesion can improve quality of life for the many women suffering from this chronic and painful condition. Targeting the EV-associated components involved in the establishment of the pro-endometriotic niche, and consequently reducing the chronic inflammatory state, may be a promising endometriosis treatment strategy. Since EVs, as intracellular communicators in endometriosis, are involved in many immunosuppressive pathways, inhibiting this communication will target multiple molecular and cellular events related to immune dysfunction and the ongoing inflammatory process within the pro-endometriotic niche and may be the most effective in the treatment of endometriosis.

## 7. Conclusions

For a long time, the link between chronic inflammatory disorders and cancer has been questioned. Evidence highlighted in our review demonstrates intriguing similarities between tumours and chronic inflammatory disorders, such as endometriosis, regarding the immunosuppressive role of EVs in the regulation of pathophysiological processes relevant in both disorders. The discussed literature points to the role of EVs as linking factors for both OvCa and endometriosis. As we have demonstrated, EVs participate in the regulation of similar immunosuppressive pathways in the tumor niche, as well as in the pro-endometriotic niche in the pelvic cavity (see the summarized data in Table 1 and Figure 2).

There is strong evidence linking endometriosis with oncological pathologies of the gynaecologic tract, in particular with OvCa [106]. Both microenvironments, including the tumor niche in OvCa as well as the pro-endometriotic niche in endometriosis, exhibit a strong chronic inflammatory and immuno-suppressive status that was shown to be regulated by EVs. Therefore, we suggest that EVs are central mediators linking the pathophysiology of both diseases, acting directly and indirectly on both immune and non-immune cells present in the disease niche. Discussed immunosuppressive mechanisms mediated by EVs in both pathologies include a macrophage polarization switch, inhibition of T-cell and NK-cell functions, facilitation of lesions development through support of stromal cell survival, growth and implementation. Since the ongoing inflammatory process within the tumor microenvironment facilitates tumorigenesis [158], it is believed that a chronic inflammatory microenvironment of the pro-endometriotic niche in endometriosis may similarly predispose to cancer formation. Moreover, endometriosis shares a possible similar genetic origin with another gynaecologic tract malignancy—endometrial carcinoma [114]. However, despite some reported premisses, whether endometrial cancer is associated with endometriosis on a clinical and molecular level is highly questionable, and further research is needed to determine if the two conditions are linked on a clinical and molecular level.

Both the tumour microenvironment [159] and the endometrial pro-endometriotic niche [120] are a complex mix of vasculature, inflammatory cells, and stromal cells that serves as a the essential “soil” for disease modulation. Given the complexities of tumor and chronic inflammatory microenvironments, it is evident that successful therapeutics will require targeting many components involved in pathology niche formation. Since EVs act as important intercellular communication tools affecting most of these components, EV-targeted therapeutic strategies will provide an opportunity for a multi-factoral approach promising best clinical effects.

The concept of personalised medicine has pushed the field of gynaecologic oncology and chronic inflammatory diseases to drill down on the genetic and proteomic changes of the EV cargo isolated from various biological fluids of patients in the hope of identifying individual therapeutical targets. Because of their accessibility, stability, and unique cargo, EVs have the potential to be used not only as therapeutic targets, but also as diagnostic and prognostic biomarkers for endometriosis and cancer. On the other hand, the use of EVs as diagnostic biomarkers or therapeutic targets is fraught with unique methodological limitations [160]. Therefore, additional analysis of EVs, in terms of the establishment of easy and precise methodologies for purifying different subpopulations of EVs and analysing their contents, will be required in for their clinical implementation in the future. Moreover, the utilisation of EVs as drug delivery vehicles or anticancer vaccines appears a promising strategy for treating gynecological malignancies, including OvCa. Because of their stability in body fluids, tropism to specific organs and minimal side effects, EVs can successfully deliver anticancer factors to target cancer cells [161]. 

While the cargo of OvCa-derived EVs has already been shown to be useful for diagnosis and treatment of OvCa, so far, the small number of relevant articles on the role of endometriosis-derived EVs and their cargo in various pathophysiological pathways related to endometriosis is a limitation of the current review. Therefore, future studies of the mechanisms regulating immunosuppressive mechanisms that drive the establishment of pro-endometriotic niche could provide valuable mechanistic insights into pathways central to the development and progression of endometriosis, as well as inspire new strategies for early diagnosis of this enigmatic disease. As there are only a few reviews on the role of EVs in endometriosis, especially with a connection to tumor development, we believe that this review will be a valuable contribution to the EV scientific field.

## Figures and Tables

**Table 1:** A detailed summary of EV-mediated immunosuppressive mechanisms in ovarian cancer, endometrial cancer and endometriosis.

Disease	Source of EVs	Studied Cargo	Cargo Type	Mechanism	Target Cells	References
Ovarian Cancer	Serum	FasL	protein	Apoptosis, down-regulation of CD3zeta	Jurkat T-cells	[24]
	Ascites	FasL	protein	Apoptosis, down-regulation of CD3zeta and JAK-3	Jurkat T-cells	[25]
	Ascites	GD3	glycosphingolipid	NKTs arrest by competing with natural ligands for CD1b binding	NKTs	[27]
	OvCa cells (high grade serous and endometroid carcinoma)	plasma gelsolin	protein	Apoptosis of CD8+ T-cells via FLIP downregulation and CASP-3 activation, polarization of CD4+ T-cells towards Th2	T-cells	[29]
	Ascites	phosphatidylserine	phospholipid	Blocking of T-cell activation by inhibiting NF-kb/NFAT signaling	CD8+ T-cells	[30]
	OvCa cells (ovarian endometroid adenocarcinoma), serum	circ-0001068	circRNA	T-cell exhaustion and PD-1 induction by competing with mir-28-5p	CD8+ T-cells	[31]
	Ascites	Singlec-10	protein	Blocking T-cell activation	CD8+ T-cells	[35]
	Ascites	not specified	not specified	Proliferation arrest, down-regulation of CD69, CD107a, cytokine production	CD8+ T-cells	[37]
	OvCa cells (adenocarcinoma), serum, ascites	ARG1	protein	Proliferation arrest, down-regulation of CD3zeta	CD8+ and CD4+T-cells	[42]
	OvCa cells (epithelial ovarian carcinoma)	NKG2D ligands	protein	Down-regulation of NKG2D receptors	NK-cells	[44]
	OvCa cells (epithelial ovarian carcinoma)	not specified		M2 polarization through the (SOCS)4/5/STAT3 pathway	Macrophages	[48]
	OvCa cells (epithelial ovarian carcinoma)	miR-940	miRNA	M2 polarization	Macrophages	[49]
	OvCa cells (epithelial ovarian carcinoma)	miR-222-3p	miRNA	M2 polarization by inducing STAT3 expression	Macrophages	[50]
	OvCa cells (serous adenocarcinoma, serous cystadenocarcinoma, endometrioid adenocarcinoma)	miR-1246	miRNA	M2 polarization by down-regulation of Cav1, chemoresistance	Macrophages, OvCa cells	[51]
	OvCa cells (epithelial ovarian carcinoma)	CD47	protein	Decreased tumor-infiltration phagocytosis by M1 macrophages	Macrophages	[52]
	Ascites	TGFβ, IL-10	protein	Induction of Tregs, enhancement of Treg suppressive function	Tregs	[55]
	Urine	HSP70	protein	Activation of mdscs through TLR2-binding	MDSCs	[59]
	OvCa cells (serous surface papillary adenocarcinoma, serous cystadenocarcinoma)	TGFβ	protein	Transition of normal fibroblasts into cafs	CAFs	[62]
	OvCa cells (serous cystadenocaricnoma, high grade serous adenocarcinoma)	TGFβ	protein	Generation of tumor-associated myofibroblastic cells from AT-mscs through induction PI3K/AKT signaling pathways	AT-MSCs	[63]
Endometrial cancer	EC cells (poorly differentiated grade 3 (G3) endometrial carcinoma)	miR-21	miRNA	Monocytes polarization into M2 phenotype	Monocytes	[99]
	M2 macrophages	hsa_circ_0001610	lncRNA	Enhancement of EC radioresistance	EC cells	[100]
Endometriosis	Immortalized endometriotic, ectopic epithelial cells	not specified	lncRNA	Upregulation of proinflammatory cytokine production-tnfα, upregulation of G-CSF, downregulation of MDC	Endothelial cells	[128]
	eESCs	not specified		M2 polarization, suppression of phagocytic ability, increased M2 macrophage recruitment into the ectopic lesions	Peritoneal macrophages	[136]
	eESCs	miR-301a-3p	miRNA	M2 polarization through the PTEN/PI3Kγ signaling pathway, upregulation of Arg-1 expression on macrophages	Macrophages	[137]
	M2 macrophages	miR-223	miRNA	Naive monocyte differentiation into M2 phenotype, terminal maturation of other progenitor lineages into granulocytes or megakaryocytes, uptake by endothelial, epithelial, and fibroblast cells	Naive monocytes, progenitor cells, endothelial, epithelial, and fibroblast cells	[139]
	Peritoneal macrophages	miR-22-3p	miRNA	Increase of proliferation and migration in eesc via SIRT1/NF-kβ pathway	eESCs	[132]
	Peritoneal macrophages	CHL1-A	lncRNA	Promotion of proliferation, migration, and invasion of eescs, and inhibition of their apoptosis via downregulating mir-610 and upregulating MDM2	eESC	[131]
	Endometrioma aspirates	CD39 and CD73	protein	Inhibition T-cell and NK-cell response, promotion of Treg proliferation and upregulation of CTLA-4 and PD-1 expression	T-cells, NK-cells, Treg cells	[143]

Three forms of non-coding RNA were recognized in the disease conditions: miRNA, microRNA; lncRNA, long noncoding RNA; circRNA, circular RNA. Abbreviations: AKT1, serine/threonine kinase 1; ARG1, arginase 1; AT-MSCs, adipose tissue-derived mesenchymal stem cells; CAFs, cancer-associated fibroblasts; CASP-3, caspase 3; Cav1, caveolin-1; CD107a, lysosome-associated membrane protein-1; CD39, ecto-nucleoside triphosphate diphosphohydrolase 1; CD3zeta, cluster of differentiation 3zeta; CD4+ T-cells, helper T lymphocytes; CD47, cluster of differentiation 47; CD69, cluster of differentiation 69; CD73, ecto-5′-nucleotidase;CD8+ T-cells, cytotoxic T lymphocytes; CTLA-4, cytotoxic T lymphocyte antigen 4; EC, endometrial cancer; eESCs, ectopic endometrial stromal cells; FasL, Fas ligand; FLIP, flice inhibitory protein; G-CSF, granulocyte colony-stimulating factor; GD3, ganglioside precursor disialohematoside; HSP70, heat shock protein 70; IL-10, interleukin 10; JAK-3, Janus kinase 3; MDC, macrophage-derived chemokine; MDM2, mouse double minute 2 homolog; MDSCs, myeloid-derived suppressor cells; MSCs, mesenchymal stem cells; NFAT, nuclear factor of activated T-cells; NF-kβ, nuclear factor kappa-light-chain-enhancer of activated B lymphocytes; NK-cells, natural killer cells; NKG2D, natural killer group 2D; NKTs, natural killer T-cells; OvCa, ovarian cancer; PD-1, programmed cell death protein 1; PI3K, phosphoinositide 3-kinase; SIRT1, silent information regulator sirtuin 1; SOCS, suppressor of cytokine signaling; STAT3, signal transducer and activator of transcription 3; T-cells, T lymphocytes; TGFβ, transforming growth factor beta;Th2, T helper cell type 2; TLR2, toll-like receptor 2; TNF-α, tumor necrosis factor-alpha; Tregs, regulatory T lymphocytes.

## Data Availability

Not applicable.
